# Direct ultrasensitive electrochemical biosensing of pathogenic DNA using homogeneous target-initiated transcription amplification

**DOI:** 10.1038/srep18810

**Published:** 2016-01-05

**Authors:** Yurong Yan, Shijia Ding, Dan Zhao, Rui Yuan, Yuhong Zhang, Wei Cheng

**Affiliations:** 1 The center for Clinical Molecular Medical detection, The First Affiliated Hospital of Chongqing Medical University, Chongqing 400016, PR China; 2 Key Laboratory of Laboratory Medical Diagnostics (Ministry of Education of China), Department of Laboratory Medicine, Chongqing Medical University, Chongqing 400016, PR China

## Abstract

Sensitive and specific methodologies for detection of pathogenic gene at the point-of-care are still urgent demands in rapid diagnosis of infectious diseases. This work develops a simple and pragmatic electrochemical biosensing strategy for ultrasensitive and specific detection of pathogenic nucleic acids directly by integrating homogeneous target-initiated transcription amplification (HTITA) with interfacial sensing process in single analysis system. The homogeneous recognition and specific binding of target DNA with the designed hairpin probe triggered circular primer extension reaction to form DNA double-strands which contained T7 RNA polymerase promoter and served as templates for in vitro transcription amplification. The HTITA protocol resulted in numerous single-stranded RNA products which could synchronously hybridized with the detection probes and immobilized capture probes for enzyme-amplified electrochemical detection on the biosensor surface. The proposed electrochemical biosensing strategy showed very high sensitivity and selectivity for target DNA with a dynamic response range from 1 fM to 100 pM. Using *salmonella* as a model, the established strategy was successfully applied to directly detect *invA* gene from genomic DNA extract. This proposed strategy presented a simple, pragmatic platform toward ultrasensitive nucleic acids detection and would become a versatile and powerful tool for point-of-care pathogen identification.

Nucleic acid testing for pathogens plays essential role in rapid pathogen identification and diagnosis of infectious diseases, especially for applications in the point-of-care setting where fast turnaround times are required and centralized laboratory facilities are unavailable^1^,^2^. The development of the polymerase chain reaction (PCR) has given rise to powerful methods for the rapid and accurate diagnosis of infectious disease. However, PCR-based techniques, requiring high-precision instruments for thermal cycling amplifications and stringent laboratory compartmentalization for prevention of false positive results, can only be used in centralized laboratories of hospitals^1^,^2^,^3^. Thus, integrating nucleic acid amplification with signal detection in a high-effective, robust, and user-friendly format towards point-of-care applications remains challenging.

Recently, biosensor-based system has attracted considerable attention for application in nucleic acids analysis due to its miniaturization, convenient automatization, rapid response, and multianalysis capability^4^,^5^. Progressive advances in isothermal amplification chemistries, an alternative to PCR for nucleic acid amplification, are paving the way for the development of high sensitive DNA biosensors owing to their high efficiency and rapid amplification kinetics under isothermal conditions^6^,^7^. Various isothermal amplification strategies such as rolling circle amplification (RCA)^8^,^9^,^10^, loop-mediated amplification (LAMP)^11^, nicking enzyme signal amplification (NESA)^12^, exonuclease III-aided signal amplification^13^,^14^, strand displacement reaction (SDR)^15^,^16^ have been integrated in biosensing systems to explore the excellent detection capability toward nucleic acids and other biomacromolecules. However, these isothermal amplification-based biosensors are mostly designed to implement target binding and signal amplification on the interface of biosensors, which is necessarily accompanied by the relatively low binding efficiency and enzyme kinetics owing to the steric hindrance, variant chemical microenvironment and surface crowding effect at the biosensor surface^17^,^18^,^19^. These defects of heterogeneous target binding and amplification maybe show little impact on the analysis of synthetic target DNA, but inevitably compromise the sensitivity and reproducibility of the biosensors, especially for direct detection a low abundance target gene from complicated genomic DNA in real sample^20^. Interfacial engineering with nanostructure have been proposed to obtain improved target recognition efficiency and upgraded detection sensitivity^21^,^22^. Unfortunately, the variability of surface micro-/nano-fabrication often affected the reproducibility and quantification of target in complicated matrix^23^. Therefore, the development of pragmatic and simple biosensing methodology for highly sensitive and direct detection pathogenic gene is still highly desirable for rapid pathogen identification.

DNA-dependent RNA polymerase, such as T7 RNA polymerase, could recognize the specific promoter sequence and transcribe its downstream DNA sequence. Compared with isothermal DNA amplification such as RCA, SDA, the transcription amplification based on T7 polymerase holds higher flexibility for the design of amplification products and is highly compatible to target-dependent signal amplification. So, T7-based transcription amplification has been attempted to develop new isothermal amplification strategy for homogeneous detection of cell surface molecules, DNA binding proteins and nucleic acids^24^,^25^,^26^,^27^.

Herein, inspired by the unique property of T7-based transcription amplification, a novel homogeneous target-initiated transcription amplification (HTITA) strategy was proposed to integrate homogeneous target DNA binding and cascade signal amplification with heterogeneous electrochemical biosensing in single analysis system for ultrasensitive and specific detection of pathogenic DNA. The recognition and binding of target DNA with the designed multifunctional hairpin probe in homogeneous solution ensured uniform reaction kinetics, high binding affinity and excellent specificity. Meantime, the cascade signal amplification of the designed circular primer extension, T7 polymerase mediated RNA transcription and interfacial enzyme-amplified electrochemical readout resulted in ultra-sensitivity for target DNA detection. The *invA* gene, a highly conserved gene and potential biomarker for *Salmonella*^28^,^29^, was used as a model target to verify the proposed strategy. The proposed method shows very high sensitivity and selectivity and has been successfully applied to directly detect the *invA* gene from genomic DNA extract of *salmonella*. Thus, this work provided a simple, pragmatic platform toward ultrasensitive nucleic acids detection and would become a versatile and hopeful tool for point-of-care pathogen identification.

## Results

### Design of the biosensing strategy

**Figure 1:**
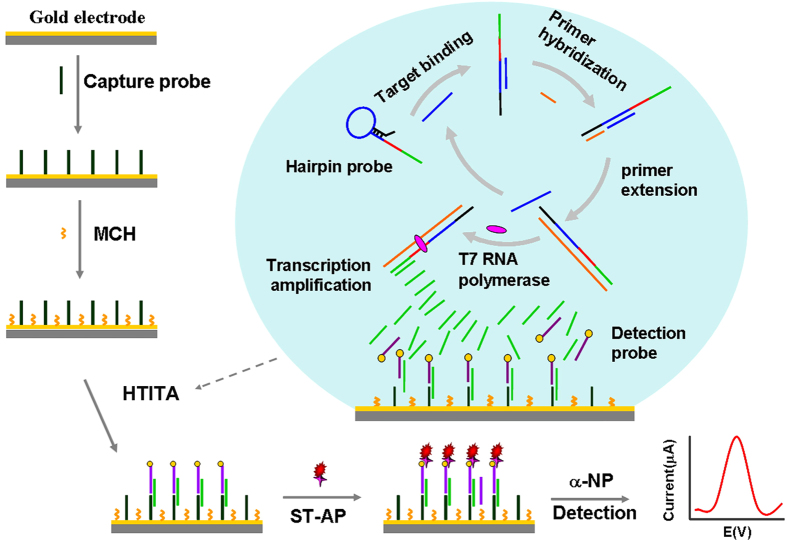
Schematic representation of the electrochemical biosensor based on HTITA for DNA detection.

The HTITA strategy and proposed electrochemical DNA biosensor are conceptually depicted in Fig. 1. The sequences of probes used in the experiments are listed in Table S1 in Supporting Information. The designed multifunctional hairpin probe contained the complementary sequences for primer hybridization, the target binding region, the T7 polymerase promoter and the transcription template. The target binding region could form loop-stem structure with the primer complementary sequences to block the primer hybridization. The specific recognization and binding of the target DNA in homogeneous solution changed the conformation of hairpin probe and opened the primer hybridization site. The hybridization of the primer triggered a primer extension reaction in the presence of Klenow Fragment of DNA polymerase I (KF exo-) and dNTPs. The target DNA was then released by the replication of DNA duplex, and bound with other hairpin probe, which led to new primer extension. So, the process of target binding, primer extension, target releasing and repeated binding was cyclic run to produce a lot of DNA duplex. Every DNA duplex product of circular primer extension naturally contained a T7 promoter and a downstream transcription template. Then, T7 RNA polymerases recognized these T7 promoter sequences as a start site for sustained transcription downstream DNA template^30^,^31^,^32^. The designed HTITA process eventually could transfer single homogeneous target-binding event into numerous single-stranded RNA products which synchronously hybridized with the biotinylated detection probes and immobilized capture probes on the biosensor surface. Streptavidin-alkaline phosphatase (ST-AP) was then labeled on the hybridized product by the specific recognition between streptavidin and biotin. Then the differential pulse voltammetric (DPV) measurement was performed to read out the oxidation of a-naphthyl which was the AP-catalyzed product of α-naphthyl phosphate (α-NP), for ultrasensitive and specific detection of pathogenic gene.

### Characterization of biosensor fabrication

**Figure 2:**
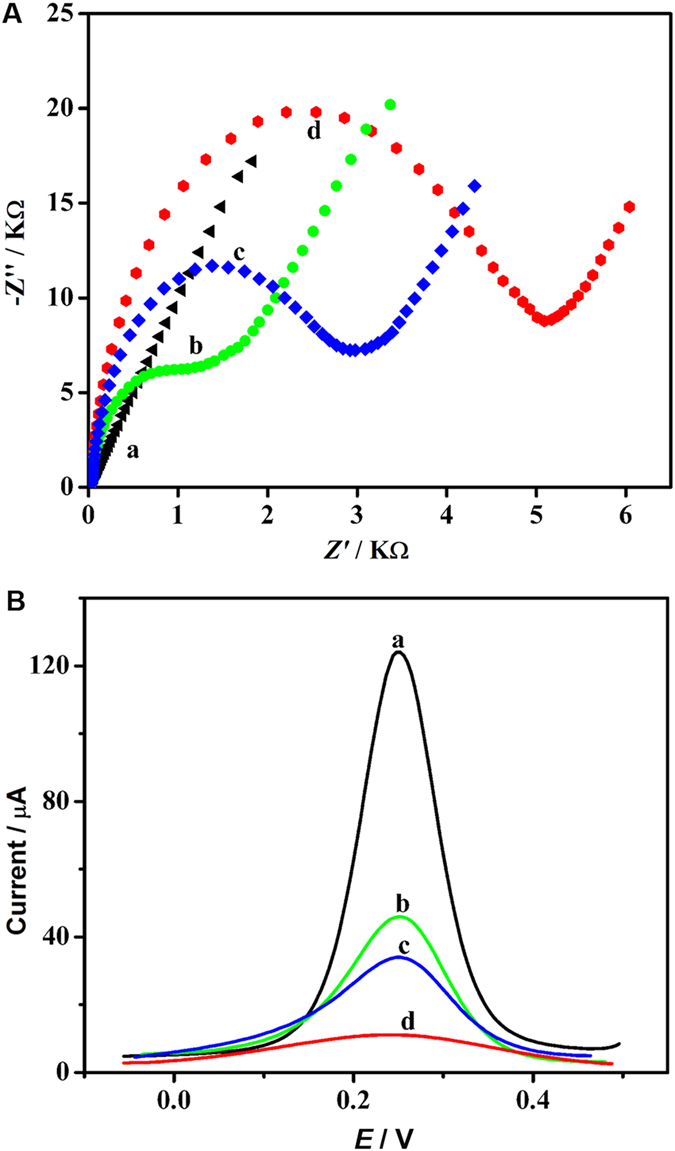
(**A**) EIS and (**B**) SWVs in 0.4 M KCl containing 0.5 mM [Fe(CN)_6_]^3−/4−^ at bare electrode (a), capture probe modified electrode (b), capture probe/MCH/salmon sperm DNA/BSA modified electrode (c), and capture probe/MCH/salmon sperm DNA/BSA modified electrode after hybridization with RNA products and detection probe (d).

Electrochemical impedance spectroscopy (EIS) and square wave voltammetry (SWV) were employed to characterize the stepwise modified processes of the biosensor. The EISs of [Fe(CN)_6_]^3−/4−^ solution at different modified electrodes were shown in Fig. 2A and the semicircle diameter of EIS curve equaled the electron transferring resistance (Ret). The bare gold electrode exhibited a nearly straight line (curve a), reflecting a mass diffusion limiting step of the electron-transfer process. The Ret increased after the capture probe was assembled on the gold electrode (GE) (curve b), suggesting that the repellence of [Fe(CN)_6_]^3−/4−^ was increased due to the negatively charged phosphate backbone of the capture probe. The Ret further enhanced after the residual active sites on GE surface were blocked with 6-mercapto-1-hexanol (MCH), bovine serum albumin (BSA) and salmon sperm DNA (curve c). This might be attributed to the electron obstruction of the hydrophobic proteins on the surface of electrode. After the prepared biosensor participated the HTITA reaction, a dramatically increase of the Ret was achieved (curve d), owing to the formation of sandwich nucleic acid structure based on the hybridization of HTITA products, detection probe and the capture probe on the biosensor surface. These results were in a good agreement with those obtained from SWV (Fig. 2B), in which the peak currents decreased upon the assembly and hybridization process on the biosensor surface. Both the EIS and SWV demonstrated successful fabrication of the biosensor and effective integration of HTITA and heterogeneous biosensing process.

### Verification of HTITA Reaction

**Figure 3:**
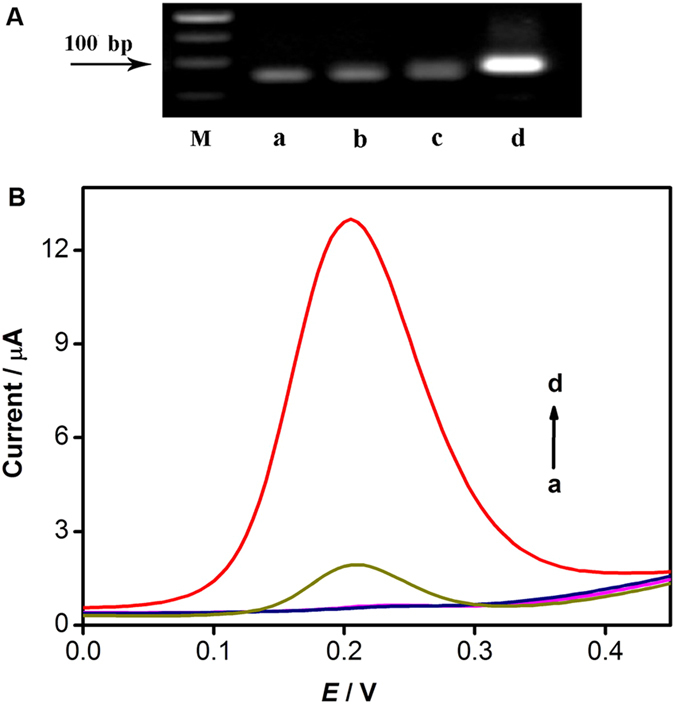
(**A**) Gel electrophoresis analysis of 1 μM hairpin probe (a), 1 μM hairpin probe and 0.1 μM target DNA without (b) and with (c) KF exo-/dNTPs, and (d) 1 μM hairpin probe, 0.1 μM target DNA and 1 μM primer with KF exo-/dNTPs after incubation for 1 h at 37 °C. (**B**) DPV curves of designed biosensor responding to 100 pM target DNA without KF exo- (a), T7 RNA polymerase (b), and 0 (c) and 100 pM (d) target DNA with KF exo- and T7 RNA polymerase, respectively.

Agarose gel electrophoresis analysis was performed to characterize target binding of designed hairpin probe and circular primer extension reaction (Fig. 3A). Compared with simple hairpin probe, the mix of hairpin probe and target DNA exhibited a slightly wide band. A further wide band was found after the mix was incubated with KF exo- and dNTPs, owing to the hybridization of hairpin probe with target DNA and resulting KF exo- induced polymerization reaction. When the primer was added to the system, a significantly light band with relative less migration exhibited, indicating a lot of DNA duplex was generated by the circular primer extension reaction in the presence of KF exo- and dNTPs. The results of gel electrophoresis analysis confirmed the reasonable design of hairpin probe and that the target-binding of hairpin probe could successfully trigger a circular primer extension reaction.

After implementing the HTITA and heterogeneous hybridization reaction, enzyme-amplified electrochemical biosensing was performed. As shown in Fig. 3B, there was no detectable DPV peak of designed biosensor corresponding to 100 pM target DNA without KF exo- or T7 RNA polymerase. Meanwhile, the DPV curves corresponding to 100 pM target DNA with HTITA exhibited a significant oxidation peak which corresponded to the oxidation of the AP-catalyzed product of α-NP. The results demonstrated the feasibility of the designed HTITA strategy and a sensitive methodology for target DNA quantitation. A slight DPV signal for blank was also observed, which was likely attributed to a little part of the transcription reaction owe to a weak hybridization of hairpin probe with primer.

### Optimization of experimental conditions

**Figure 4:**
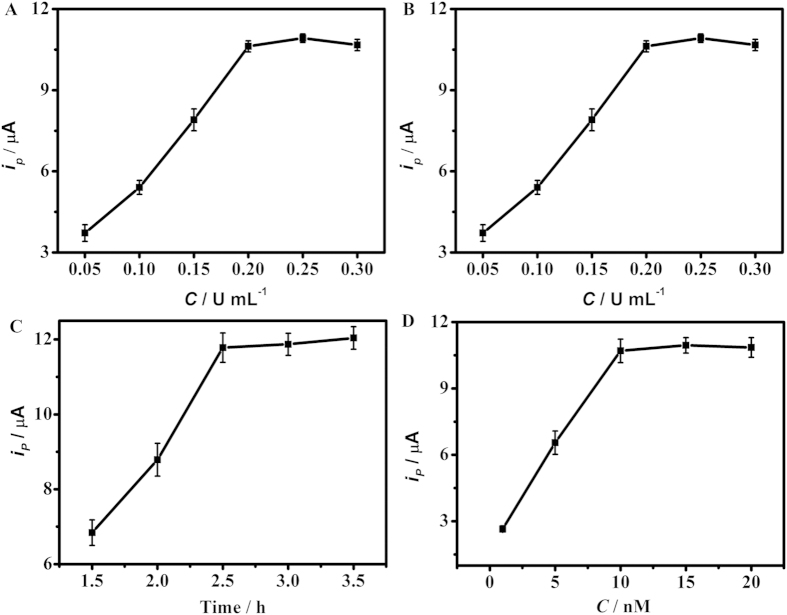
Dependences of DPV peak currents responding to 100 pM target DNA on (A) KF exo- concentration, (**B**) T7 RNA polymerase concentration, (C) HTITA reaction time and (D) detection probe concentration. When one parameter changed the others were under their optimal conditions. The error bars represent the standard deviations in three different measurements for each concentration.

In order to achieve optimal assay performance, several experimental parameters concerning HTITA process were investigated. The concentration of KF exo- and T7 RNA polymerase significantly influenced the efficiency of circular primer extension reaction and transcription amplification, respectively. As shown in Figs 4A and 4B, the peak current increased with the increasing polymerase concentrations and tended to a steady value after 0.2 U μL^−1^ of KF exo- and 0.8 U μL^−1^ of T7 RNA polymerase, respectively. So 0.2 U μL^−1^ of KF exo- and 0.8 U μL^−1^ of T7 RNA polymerase were chosen as the optimal polymerase concentrations. A longer reaction time of HTITA could generate more RNA products for heterogeneous electrochemical biosensing, so the DPV response increased with the increasing reaction time (Fig. 4C). At 2.5 h the reaction reached saturation, which led to a constant peak current. Thus 2.5 h was used for the HTITA process.

RNA products of HTITA were synchronously hybridized with the biotinylated detection probes and immobilized capture probes on the surface of the biosensor. A suitable concentration of biotinylated detection probe was important to ensure sufficient hybridization and reduce nonspecific signal. With the increasing concentration of biotinylated detection probe the DPV peak current increased and then tended to a constant value at the concentration of 10 nM (Fig. 4D), indicating a tendency to thorough hybridization of RNA products. So 10 nM was chosen as the optimal concentration of biotinylated detection probe.

### Analytical performance of biosensor of the designed method

**Figure 5:**
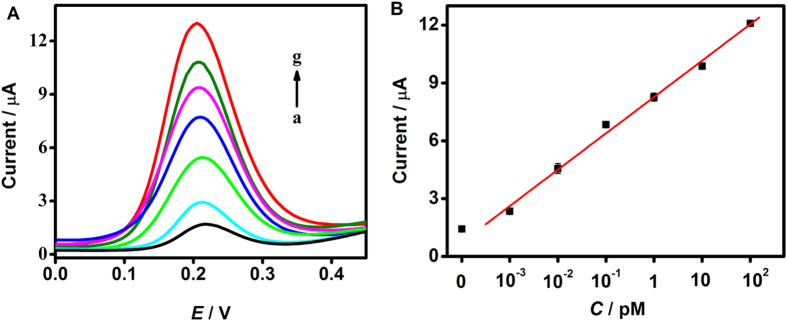
(**A**) Typical DPV curves responding to 0, 0.001, 0.01, 0.1, 1, 10, 100 pM target DNA (from a to g). (**B**) Calibration plot of DPV peak current vs. logarithm of target DNA concentration. The error bars represent the standard deviations in three different measurements for each concentration.

Under the optimal experimental conditions, the DPV peak currents increased with the increasing concentrations of target oligonucleotides (Fig. 5A). The response was proportional to the logarithm value of target DNA concentration over a 5-decade range from 1 fM to 100 pM with a linear correlation coefficient of 0.999 (Fig. 5B). The limit of detection (LOD) was calculated to be 0.97 fM in a 3σ rule. In other words, the proposed biosensor was able to respond to 580 molecules of the synthetic target DNA in a 10 μL reaction mixture. The LOD of the proposed method for detection of synthetic target DNA is comparable with those of previous biosensors based on heterogeneous target binding and DNA isothermal amplification, and is higher than those of the methods based on isothermal amplification combined with nanoparticles. But those methods based on heterogeneous target binding mostly lacked the application in real sample detection (Table 1).

To estimate the reproducibility of the developed electrochemical biosensor, the intra-assay imprecision of five different sensors at one assay and inter-assay imprecision at five different assays were examined. The intra-assay coefficients of variation (CVs) were 2.60% and 2.31% at 10 fM and 10 pM of target DNA, and the inter-assay CVs was 7.65% and 6.60% at these concentrations, respectively, indicating remarkable precision and reproducibility. The significant reproducibility was owing to uniform reaction kinetics of HTITA.

### Detection of real samples

**Figure 6:**
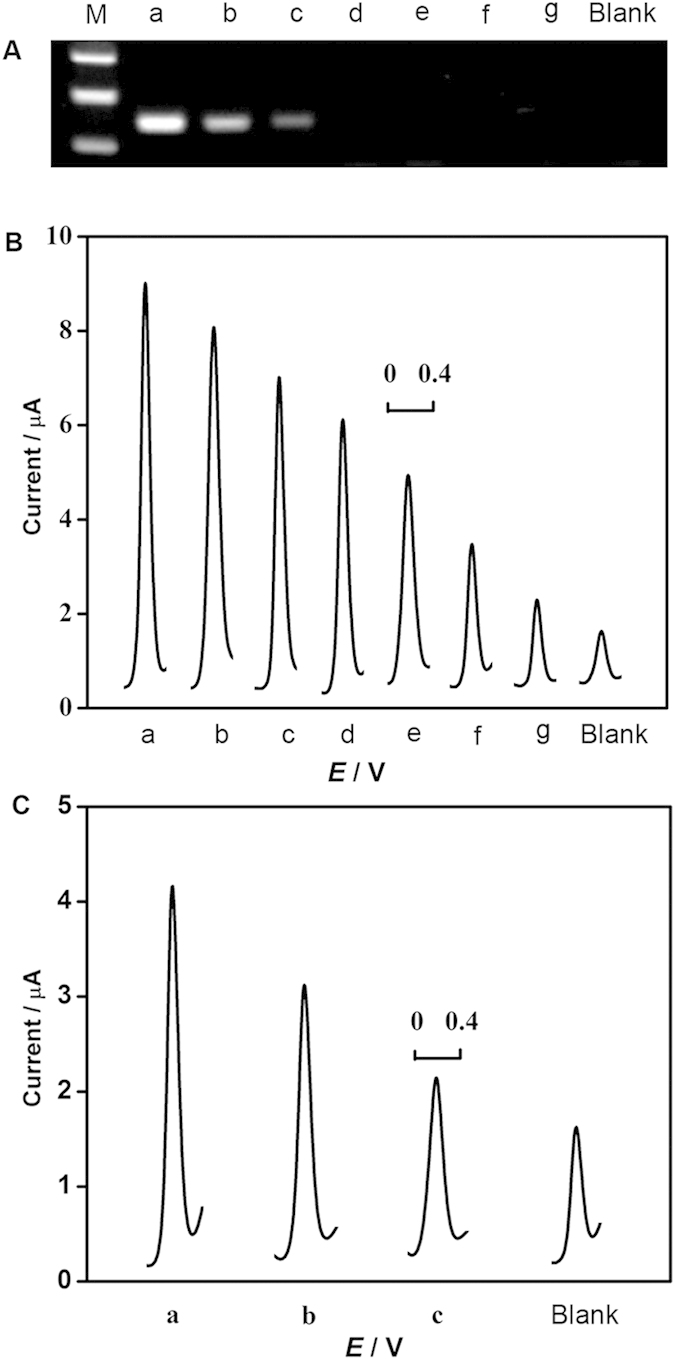
(**A**) Gel electrophoresis photos and (**B**) typical DPV curves of PCR products obtained from 10^7^, 10^6^, 10^5^, 10^4^, 10^3^, 10^2^, 10^1^ CFU mL^−1^ of *Salmonella* (from a to g) and blank, respectively. (**C**) Typical DPV curves for direct detection *invA* gene from genomic DNA extracts of 10^7^ (a), 10^6^ (b) and 10^5^ (c) CFU mL^−1^ of *Salmonella* and blank.

To evaluate the feasibility for ultrasensitive detection of nucleic acids in real samples, the established biosensor was employed to detect invA gene in genomic DNA of serially diluted *Salmonella* with and without PCR. The DPV responses for PCR products were proportional with the concentrations of *Salmonella* cells over the range of 10–10^7^ CFU mL^−1^ (Fig. 6B). The proposed strategy was further applied to directly quantitate *Salmonella* in genomic DNA extract without PCR. As shown in Fig. 6C, our established method could directly detect *Salmonella* as low as 10^5^ CFU mL^−1^. According to the detection protocol, the LOD for real samples was calculated to be about 1000 copies of *Salmonella* genomic DNA, which was comparable with that of PCR coupling with gel electrophoresis (Fig. 6A). The LOD of the proposed method for real samples were similar to that for the detection of synthetic target DNA, confirming no loss of analytical performance for detection of enormous and complicated genomic DNA in real sample and little influences of the potential interferents in the real samples on the detection accuracy.

### Specificity of the assay

**Figure 7:**
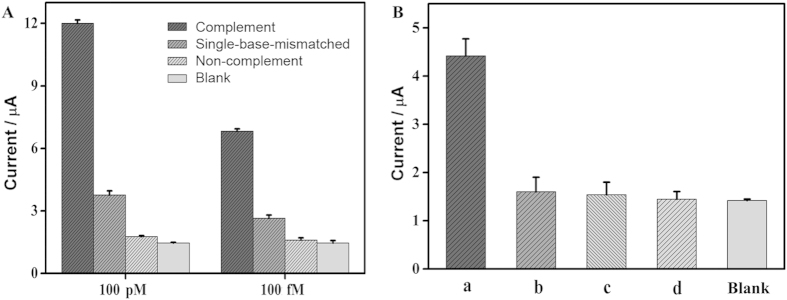
(**A**) DPV peak currents responding to 100 pM and 100 fM of target DNA, single-base-mismatched oligonucleotides, non-complementary oligonucleotides and blank, respectively. (**B**) DPV peak currents responding to genomic DNA extracts of 10^7^ CFU mL^−1^ of *Salmonella* (a), *E. coli* (b), *staphylococcus aureus* (c), *Streptococcus pneumoniae* (d) and blank. The error bars represent the standard deviations in three different measurements for each concentration.

To investigate the specificity of HTITA strategy, different DNA sequences at two concentrations of 100 pM and 100 fM were analyzed with the proposed electrochemical biosensor, respectively (Fig. 7A). The DPV responses of complementary sequence were much larger than those of single-base mismatched and non-complementary sequences, demonstrating that the designed biosensor could effectively discriminate different DNA sequences owing to the high binding specificity of hairpin probe in solution^33^. Moreover, DNA extracts of four different types of bacteria were directly analyzed with the designed method (Fig. 7B). The DPV response of *Salmonella* was obviously higher than those of *Staphylococcus aureus*, *E. Coli* and *Streptococcus pneumoniae*. The response of *Staphylococcus aureus*, *E. Coli* and *Streptococcus pneumoniae* was approximate to that of blank. These results obviously indicated that the proposed biosensor exhibited superior specificity for direct detection of pathogenic nucleic acids in real sample.

## Discussion

The ultrahigh sensitivity, specificity and the capability for real sample detection of the proposed biosensing strategy were attributed to the high efficiency of homogeneous target binding, remarkable signal amplification performance of HTITA. More importantly, the significant analytical performance was implemented in a single analysis system without the need of sophisticated instrument for temperature cycling and complicated preparation of biofunctional nano-probe or interfacial nano-fabrication, exhibiting great application potential towards point-of-care screening of pathogens. We will further explore to improve the integration of measurement process and shorten the detection time for desirable applications in the point-of-care analysis.

In summary, a novel homogeneous target-initiated transcription amplification strategy has been developed for ultrasensitive and specific electrochemical sensing of pathogenic DNA by coupling homogeneous target recognition, cascade signal amplification with interfacial sensing process in single analysis system. The designed biosensor demonstrates high sensitivity, remarkable specificity and reproducibility. Also, this assay has been successfully applied to directly detect pathogenic gene in real sample. Using the mode of spatial discrimination, the strategy has the potential for multiple and high-throughput analysis of nucleic acids. Thus the developed strategy presents a simple and pragmatic platform toward ultrasensitive DNA nucleic acids detection and would become a potential and versatile tool for point-of-care nucleic acids testing in the area of clinical diagnosis and therapy, pathogen identification and environmental monitoring in the future.

## Methods

### Preparation of genomic DNA and PCR amplification

*Salmonella typhimurium* strains used in the experiments were obtained from Chongqing Municipal Center for Disease Control and Prevention. The pure strain was grown in Luria-Bertani medium at 37 °C for 16 h with shaking. The culture was then washed twice with sterile ultrapure water by centrifugation at 12,000 rpm for 10 min and resuspended in sterile ultrapure water. Viable counts were conducted by plating 100 μL of appropriate 10-fold dilutions in sterile ultrapure water onto plate count agar in triplicate and incubating the plate at 37 °C for 24 h. The culture colonies on the plates were counted to estimate the number of viable cells in CFU mL^−1^. Genomic DNA was extracted from 1 mL of different concentration culture using the TaKaRa MiniBEST Universal Genomic DNA Extraction Kit Ver.5.0 according to the instructions and resuspended in 100 μL of sterile ultrapure water. All prepared DNA were stored at −20 °C for further use.

The PCR reactions were carried out in a total volume of 50 μL containing 5.0 μL of genomic DNA, 1.0 μL of 20 μM each primer, 25 μL of Premix Taq and 18 μL of distilled water. The cycling conditions were as follows: 35 cycles of 95 °C for 30 s, 51 °C for 30 s, and extension at 72 °C for 1 min, and a final extension at 72 °C for 4 min. PCR products were determined by running 10 μL of PCR mixtures in 2% agarose gel for 30 min and observed under ultraviolet imaging system.

### Preparation of electrochemical biosensor

The bare gold electrode was polished with 0.05 mm alumina slurries and ultrasonically treated in ultra-pure water for three times, followed by soaking in piranha solution (H_2_SO_4_:H_2_O_2_=3:1) for 10 min to eliminate other substances. After rinsed with deionized water and drying at room temperature, 10 μL of 1 μM thiolated capture probes were dropped on the pretreated electrode surface and incubated overnight at 4 °C. The resulting electrode was then washed with washing buffer, and immersed into 1 mM MCH solution for 1 h to occupy the left bare sites on electrode surface and obtain well-aligned DNA monolayer. After rinsed with washing buffer, the electrode was further treated in 0.1 M pH 7.5 Tris-HCl buffer containing 125 mg mL^−1^ salmon sperm DNA and 2% BSA for 30 min to block the nonspecific binding sites on its surface. The obtained sensor was prepared for the following DNA electrochemical measurements.

### Nucleic acids detection with proposed protocol

Prior to use, the hairpin probe was annealed for 10 min at 95 °C in a water bath and then cooled down slowly at room temperature in order to form absolute loop-stem structure. In a typical experiment, the HTITA reaction mixture contained 100 nM hairpin probe, 100 nM primer, 1 mM dNTPs, 1 mM rNTPs, 10 nM biotinylated detection probe, 0.2 U^−1^ μL KF exo-, 0.8 U μL^−1^ T7 RNA polymerase, 0.8 U μL^−1^ RNase inhibitor and 1 μL DNA sample in 10 μL 1 × T7 transcription buffer. The HTITA reaction mixture was dropped on the above prepared biosensor and incubated at 37 °C for 2.5 h to perform HTITA reaction. Then, the biosensor was further incubated at 4 °C for 0.5 h to strengthen the hybridization on the surface of biosensor.

After the HTITA reaction was completed, the biosensor was washed with washing buffer, reacted with 10 μL of 1.25 mg mL^−1^ ST-AP in DEA buffer at room temperature for 30 min. Finally, the obtained biosensor was washed with DEA buffer containing 0.05% Tween 20 thoroughly. DPV measurement was then performed in DEA buffer containing 1 mg mL^−1^ of α-NP, with modulation time of 0.05 s, interval time of 0.017 s, step potential of 5 mV, modulation amplitude of 70 mV and potential scan from 0 to +0.6 V.

## Additional Information

**How to cite this article**: Yan, Y. *et al.* Direct ultrasensitive electrochemical biosensing of pathogenic DNA using homogeneous target-initiated transcription amplification. *Sci. Rep.* **6**, 18810; doi: 10.1038/srep18810 (2016).

## Supplementary Material

Supplementary Information

## Figures and Tables

**Table 1:** Comparison between the proposed HTITA strategy and other reported electrochemical bisoensors based on heterogeneous target binding for DNA detection.

Signal amplification method	Linear range (M)	Detection limit (M)	Real sample Detection	References
Target recycling and RCA	10^−11^ ~ 10^−15^	2.5 × 10^−16^	No	9
RCA and QDs tag	10^−11^ ~ 10^−17^	11 × 10^−17^	No	10
Enzymatic cleavage	10^−10^ ~ 10^−14^	10^−14^	No	12
Exonuclease III	10^−9^ ~ 10^−14^	8.7 × 10^−15^	Yes (with PCR)	13
CSD and Au NPs	10^−12^ ~ 10^−16^	3 × 10^−17^	No	16
HTITA	10^−10^ ~ 10^−15^	9.7 × 10^−16^	Yes	This wok

## References

[b1] Niemz A., Ferguson T. M. & Boyle D. S. Point-of-care nucleic acid testing for infectious diseases. Trends Biotechnol. 29, 240–250 (2011). 21377748 10.1016/j.tibtech.2011.01.007 PMC3746968

[b2] de-Paz H. D., Brotons P. & Munoz-Almagro C. Molecular isothermal techniques for combating infectious diseases: towards low-cost point-of-care diagnostics. Expert Rev. Mol. Diagn. 14, 827–843 (2014). 25052202 10.1586/14737159.2014.940319 PMC7103708

[b3] Park J. S., Cho D. H., Yang J. H., Kim M. Y., Shin S. M. *et al.* Usefulness of a rapid real-time PCR assay in prenatal screening for group B streptococcus colonization. Ann. Lab. Med. 33, 39–44 (2013). 23301221 10.3343/alm.2013.33.1.39 PMC3535195

[b4] Rosario R. & Mutharasan R. Nucleic acid electrochemical and electromechanical biosensors: a review of techniques and developments. Rev. Anal. Chem. 33, 213–230 (2014).

[b5] Singh R., Das Mukherjee M., Sumana G., Gupta R. K., Sood S. *et al.* Biosensors for pathogen detection: A smart approach towards clinical diagnosis. Sensor. Actuat. B-Chem. 197, 385–404 (2014).

[b6] Craw P. & Balachandran W. Isothermal nucleic acid amplification technologies for point-of-care diagnostics: a critical review. Lab Chip. 12, 2469–2486 (2012). 22592150 10.1039/c2lc40100b

[b7] Gill P. & Ghaemi A. Nucleic acid isothermal amplification technologies–a review. Nucleos. Nucleot. Nucl. 3, 224–243 (2008). 10.1080/15257770701845204 18260008

[b8] Cheng W., Yan F., Ding L., Ju H. X. & Ying Y. B. Cascade signal amplification strategy for subattomolar protein detection by rolling circle amplification and quantum dots tagging. Anal. Chem. 82, 3337–3342 (2010). 20345087 10.1021/ac100144g

[b9] Wang Q., Yang C. Y., Xiang Y., Yuan R. & Chai Y. Q. Dual amplified and ultrasensitive electrochemical detection of mutant DNA Biomarkers based on nuclease-assisted target recycling and rolling circle amplifications. Biosens. Bioelectron. 55, 266–271 (2014). 24393655 10.1016/j.bios.2013.12.034

[b10] Ji H. X., Yan F., Lei J. P. & Ju H. X. Ultrasensitive electrochemical detection of nucleic acids by template enhanced hybridization followed with rolling circle amplification. Anal. Chem. 84, 7166–7171 (2012). 22823454 10.1021/ac3015356

[b11] Hsieh K., Patterson A. S., Ferguson B. S., Plaxco K. W. & Soh H. T. Rapid, Sensitive, and Quantitative Detection of Pathogenic DNA at the Point of Care through Microfluidic Electrochemical Quantitative Loop-Mediated Isothermal Amplification. Angew. Chem. Int. Ed. 51, 4896–4900 (2012). 10.1002/anie.201109115 PMC3509743 22488842

[b12] Liu S. F., Liu T. & Wang L. Label-free, isothermal and ultrasensitive electrochemical detection of DNA and DNA 3′-phosphatase using a cascade enzymatic cleavage strategy. Chem. Commun. 51, 176–179 (2015). 10.1039/c4cc08140d 25387573

[b13] Luo C. H., Tang H., Cheng W., Yan L., Zhang D. C. *et al.* A sensitive electrochemical DNA biosensor for specific detection of Enterobacteriaceae bacteria by Exonuclease III-assisted signal amplification. Biosens. Bioelectron. 48, 132–137 (2013). 23669045 10.1016/j.bios.2013.03.084

[b14] Liu S. F., Wang C. F., Zhang C. X., Wang Y. & Tang B. Label-free and ultrasensitive electrochemical detection of nucleic acids based on autocatalytic and exonuclease III-assisted target recycling strategy. Anal. Chem. 85, 2282–2288 (2013). 23320625 10.1021/ac303225p

[b15] Cheng W., Ding S. J., Li Q., Yu T. X., Yin Y. B. *et al.* A simple electrochemical aptasensor for ultrasensitive protein detection using cyclic target-induced primer extension. Biosens.Bioelectron. 36, 12–17 (2012). 22538057 10.1016/j.bios.2012.03.032

[b16] Gao F. L., Zhu Z., Lei J. P., Geng Y. & Ju H. X. Sub-femtomolar electrochemical detection of DNA using surface circular strand-replacement polymerization and gold nanoparticle catalyzed silver deposition for signal amplification. Biosens. Bioelectron. 39, 199–203 (2013). 22883748 10.1016/j.bios.2012.07.035

[b17] Vijayendran R. A. & Leckband D. E. A quantitative assessment of heterogeneity for surface-immobilized proteins. Anal. Chem. 73, 471–480 (2001). 11217749 10.1021/ac000523p

[b18] Miranda-Castro R., Marchal D., Limoges B. & Mavre F. Homogeneous electrochemical monitoring of exonuclease III activity and its application to nucleic acid testing by target recycling. Chem. Commun. 48, 8772–8774 (2012). 10.1039/c2cc34511k 22829093

[b19] Zhang H., Li F., Dever B., Li X. F. & Le X. C. DNA-mediated homogeneous binding assays for nucleic acids and proteins. Chem. Rev. 113, 2812−2841 (2013). 23231477 10.1021/cr300340p

[b20] Hao F. N., Xu Y., Chang Z., Xing R., Wang Q. J. *et al.* A non-immobilizing electrochemical DNA sensing strategy with homogenous hybridization based on the host–guest recognition technique. Biosens. Bioelectron. 26, 2655–2659 (2011). 20346643 10.1016/j.bios.2010.02.021

[b21] Wen Y., Pei H., Shen Y. & Xi J., Lin M. *et al.* DNA nanostructure-based interfacial engineering for PCR-free ultrasensitive electrochemical analysis of microRNA. Sci. Rep. 2, srep00867 (2012). 10.1038/srep00867 PMC3499757 23162691

[b22] Kelley S. O., Mirkin C. A., Walt D. R., Ismagilov R. F., Toner M. *et al.* Advancing the speed, sensitivity and accuracy of biomolecular detection using multi-length-scale engineering. Nat. Nanotechnol. 9, 969–980 (2014). 25466541 10.1038/nnano.2014.261 PMC4472305

[b23] Zhang H. Q., Zhao Q., Li X. F. & Le X. C. Ultrasensitive assays for proteins. Analyst. 132, 724–737 (2007). 17646870 10.1039/b704256f

[b24] Zhao J., Zhang L., Chen C., Jiang J. & Yu R. A novel sensing platform using aptamer and RNA polymerase-based amplification for detection of cancer cells. Anal. Chim. Acta 745, 106–111 (2012). 22938613 10.1016/j.aca.2012.07.030

[b25] Yu C. Y., Yin B. C., Wang S., Xu Z. & Ye B. C. Improved ligation-mediated PCR method coupled with T7 RNA polymerase for sensitive DNA detection. Anal. Chem. 86, 7214–7218 (2014). 25033096 10.1021/ac502275z

[b26] Ma F., Yang Y. & Zhang C. Ultrasensitive detection of transcription factors using transcription-mediated isothermally exponential amplifi cation-induced chemiluminescence. Anal. Chem. 86, 6006−6011 (2014). 24865817 10.1021/ac5017369

[b27] Yin B. C., Wu S., Ma J. L. & Ye B. C. A novel molecular beacon-based method for isothermal detection of sequence-specific DNA via T7 RNA polymerase-aided target regeneration. Biosens. Bioelectron. 68, 365–370 (2015). 25613814 10.1016/j.bios.2015.01.020

[b28] Malorny B., Hoorfar J., Bunge C. & Helmuth R. Multicenter validation of the analytical accuracy of Salmonella PCR: towards an international standard. Appl. Environ. Microbiol. 69, 290–296 (2003). 12514007 10.1128/AEM.69.1.290-296.2003 PMC152403

[b29] Daum L. T., Barnes W. J., McAvin J. C., Neidert M. S., Cooper L. A. *et al.* Real-time PCR detection of Salmonella in suspect foods from a gastroenteritis outbreak in Kerr County, Texas. J. Clin. Microbiol. 40, 3050–3052 (2002). 12149377 10.1128/JCM.40.8.3050-3052.2002 PMC120641

[b30] Zhang H. T., Kacharmina J. E., Miyashiro K., Greene M. I. & Eberwine J. Protein quantification from complex protein mixtures using a proteomics methodology with single-cell resolution. Proc. Natl. Acad. Sci. USA 98, 5497–5502 (2001). 11320219 10.1073/pnas.101124598 PMC33241

[b31] Zhang H., Cheng X., Richter M. & Greene M. I. A sensitive and high-throughput assay to detect low-abundance proteins in serum. Nat. Med. 12, 473–477 (2006). 16532003 10.1038/nm1378

[b32] Kattah M. G., Coller J., Cheung R. K., Oshidary N. & Utz P. J. HIT: a versatile proteomics platform for multianalyte phenotyping of cytokines, intracellular proteins and surface molecules. Nat. Med. 14, 1284–1289 (2008). 18849997 10.1038/nm.1755 PMC3334282

[b33] Tsourkas A., Behlke M. A., Rose S. D. & Bao G. Hybridization kinetics and thermodynamics of molecular beacons. Nucleic Acids Res. 31, 1319–1330 (2003). 12582252 10.1093/nar/gkg212 PMC150230

