# Supplementary material for: Direct ultrasensitive electrochemical biosensing of pathogenic DNA using homogeneous target-initiated transcription amplification
Source: Sci Rep. 2016 Jan 5;6:18810. doi: 10.1038/srep18810 (PMC4700466; doi:10.1038/srep18810)
Supplement: Supplementary Information [file srep18810-s1.pdf]

# Supporting Information

## Direct ultrasensitive electrochemical biosensing of pathogenic DNA using homogeneous target-initiated transcription amplification

**Yurong Yan,[1,2] Shijia Ding,[2] Dan Zhao,[2] Rui Yuan,[2] Yuhong Zhang,[1] and Wei Cheng[1]**

[1]The center for Clinical Molecular Medical detection, The First Affiliated Hospital of Chongqing Medical University, Chongqing 400016, P.R. China, [2]Key Laboratory of Laboratory Medical Diagnostics (Ministry of Education of China), Department of Laboratory Medicine, Chongqing Medical University, Chongqing 400016, P.R. China. Correspondence and requests for materials should be addressed to W. C. (email: chengwei@cqmu.edu.cn)

## Supplementary Experiment Details

**Materials and Reagents.** Streptavidin-alkaline phosphatase (ST-AP), salmon sperm DNA, bovine serum albumin (BSA), 6-mercapto-1-hexanol (MCH), and a-naphthyl phosphate (a-NP) were purchased from Sigma-Aldrich (USA). T7 RNA polymerase, Klenow Fragement (3'→5'exo-) (KF exo-), dNTP mixture (dNTPs)，rNTP mixture (rNTPs) and RNase inhibitor were obtained from Sangon Inc. (Shanghai, China). Diethylpyrocarbonate (DEPC) was obtained from Solarbio (Beijing, China). Universal Genomic DNA Extraction Kit, Premix Taq, DL500 DNA Marker and agarose were purchased from Takara (Dalian, China). The oligonucleotides were synthesized and purified by Sangon Inc. (Shanghai, China). Their sequences are listed in Table S1.

All other reagents were of analytical reagent grade. All aqueous solutions were prepared using ultrapure water (≥18MΩ, Milli-Q, Millipore). In order to protect RNA from RNase degradation, all solutions and water were treated with DEPC by mixing 0.1% of DEPC, and the mixed solutions were stored overnight prior to autoclaving. Premix Taq contained 1.25 U of DNA polymerase, 2 × Taq buffer, 0.4 mM dNTPs. 1 × T7 transcription buffer was 40 mM Tris-HCl buffer (pH 7.9) containing 10 mM NaCl, 6 mM $Mgcl_2$, 10 mM DTT and 2 mM spermidine. The washing buffer was 40

mM Tris-HCl buffer (pH 7.5) containing 10 mM NaCl, 6.0 mM $MgCl_2$ and 0.05% Tween-20. Diethanolamine (DEA) buffer (pH 9.6) contained 0.1 M diethanolamine, 1 mM $MgCl_2$, 100 mM KCl.

**Table S1. Sequences of Oligonucleotides Used in the Experiments**

| Oligonucleotide [a] | Sequence(5'- 3') [b] |
|---|---|
| Hairpin probe | TGGAGTGTGACAATGGTGTTTGTTCTATAGTGAGTCGTATTA**TAGCTACCGGCCTTCAAATCGGCA**AAA*CGGTAGCTA*CCCCC |
| Primer | TAGCTACC |
| Capture probe | SH-$(CH_2)_6$-TTTTTTTTTTTTTGGAGTGTGACAA |
| Detection probe | TGGTGTTTGTTTTTTTTT- $(CH_2)_6$ -Biotin |
| Target DNA | TGCCGATTTGAAGGCCGGTAGCTA |
| sDNA | TGCCGATTTGAGGGCCGGTAGCTA |
| nDNA | CTGGCATGGCGTGCTGTCGAGTAG |
| Forward primer | GCATCCGCATCAATAATACCG |
| Reverse primer | TTCTCTGGATGGTATGCCC |

[a] sDNA, Single-base-mismatched oligonucleotide; nDNA, Non-complementary oligonucleotide.

[b] The underlined letters in hairpin probe represent the T7 RNA polymerase promoter, the boldface letters represent the target binding region, and italicized letters indicate the complementary sequences for primer hybridization.

**Apparatus.** All electrochemical measurements were performed on a CHI660D electrochemical workstation (Shanghai Chenhua Instruments Co. Ltd., China) with a conventional three-electrode system composed of platinum wire as auxiliary, Ag/AgCl electrode as reference, and a 3-mm diameter gold electrode (GE) as working electrode. The PCR was carried out using a My Cycler thermal cycler (Bio-Rad Laboratories, USA). Gel images were recorded on an imaging system (Bio-Rad Laboratories, USA).
